# The Intuitive Eating Scale-2 Adapted for Mexican Pregnant Women: Psychometric Properties and Influence of Sociodemographic Variables

**DOI:** 10.3390/nu15224837

**Published:** 2023-11-19

**Authors:** María Eugenia Flores-Quijano, Cecilia Mota-González, Guadalupe Rozada, Jacqueline Citlalli León-Rico, María Eugenia Gómez-López, Rodrigo Vega-Sánchez

**Affiliations:** 1 Department of Nutrition and Bioprogramming, Instituto Nacional de Perinatología, Mexico City 11000, Mexico; maru_fq@yahoo.com (M.E.F.-Q.); jaki.leon94@gmail.com (J.C.L.-R.); 2 Department of Psychology, Instituto Nacional de Perinatología, Mexico City 11000, Mexico; ceciliainper@gmail.com (C.M.-G.); eugeniagomez2712@yahoo.com.mx (M.E.G.-L.); 3 Private Consultant, Alimentación Plena, Mexico City 06760, Mexico; alimentacion.plena@gmail.com

**Keywords:** eating behaviors, intuitive eating, intuitive eating scale, pregnancy, psychometric properties, sociodemographic factors

## Abstract

A weight-inclusive approach to health involves the promotion of intuitive eating, i.e., the individual’s ability to be aware of their physiological hunger and satiety cues to determine when and how much to eat, while paying attention to how certain foods affect their body. The second version of the Intuitive Eating Scale (IES-2) evaluates four interrelated traits of intuitive eating: Unconditional Permission to Eat (UPE), Eating for Physical rather than emotional Reasons (EPR), Reliance on internal Hunger/Satiety Cues (RHSC), and Body–Food Choice Congruence (BFCC). In this study, our aim was to evaluate the psychometric properties of a Mexican Spanish adaptation of the IES-2 for pregnant women and examine the relationship between intuitive eating traits and maternal sociodemographic characteristics. A sample of 514 pregnant women answered our IES-2 adaptation and a sociodemographic questionnaire. We determined the quality, validity, and reliability of our adaptation through descriptive measures, frequency distributions, intra-class correlations, and extreme answer group comparison for each item, eliminating those with weak technical properties. We then performed an exploratory principal component analysis and a confirmatory factor analysis. Last, we analyzed the association between intuitive eating and maternal sociodemographic and reproductive variables through correlation tests and multivariable linear regressions. Psychometric tests confirmed the validity and reliability of our IES-2 adaptation, which comprised 18 out of the 23 original items. Notably, both the exploratory and confirmatory factor analyses yielded not four but five factors, due to the EPR subscale splitting in two (the “emotional” and “physical” components of EPR). We attribute this novel finding to the emotional manifestations that naturally accompany pregnancy, which may incline pregnant women to base their eating behaviors more on the emotional than the physical component that would otherwise dominate their EPR trait. Further research is also needed about the UPE subscale during pregnancy, due to item removal and subtle changes in meaning. Finally, the influence of sociodemographic variables on the IES-2 score was extremely low, suggesting that other variables, possibly of a psychological nature, may have greater influence on a pregnant woman’s intuitive eating.

## 1. Introduction

For over 10 years, increasing evidence has shown several disadvantages of a weight-centered approach to health. These include preoccupation with food and body image, repeated cycles of weight loss and regain, eating disorders, fostering stigmatization in health care and society, and not accounting for wider social determinants of health, among others [1,2].

Nevertheless, during pregnancy, the nutritional approach to health is still a weight-normative one. International guidelines for perinatal care focus on pregestational body mass index (pgBMI) and the promotion of weight management through caloric restraint and physical activity as alleged means for achieving adequate perinatal health, i.e., lower risk of maternal and fetal adverse outcomes [3]. However, while diet- and physical activity-based interventions during pregnancy may achieve a short-term reduction in gestational weight gain, they do not yield the expected lower risk of maternal/fetal negative outcomes [4]. Yet they do entail the aforementioned disadvantages of a weight-centered approach to health.

In light of the shortcomings and potential risks of the weight-normative approach to health, a weight-inclusive approach has been proposed, which “considers empirically supported practices that enhance people’s health in patient care and public health settings regardless of where they fall on the weight spectrum” [2]. An important component of this approach involves the promotion of intuitive eating, i.e., the individual’s ability to be aware of their physiological hunger and satiety cues to determine when and how much to eat, while paying attention to how certain foods affect their body.

The Intuitive Eating Scale (IES) was developed as a means to evaluate the degree to which a person shows various interrelated traits that encompass intuitive eating [5]. The first version of the scale measured three such traits: (1) Unconditional Permission to Eat (UPE) shows a disposition to eat whatever food is desired at a particular moment; (2) Eating for Physical rather than emotional Reasons (EPR) reflects whether food is used to satisfy a physical hunger rather than to cope with emotional distress; and (3) Reliance on internal Hunger/Satiety Cues (RHSC) measures how much an individual is aware of their internal hunger and satiety signals and trusts these signals to guide their eating behavior. In a later version (IES-2), the scale was further refined and included a fourth trait, Body–Food Choice Congruence (BFCC), which measures the extent to which an individual matches their food choices to their body’s needs [6].

Various studies have used the first version of the IES with pregnant women [7,8,9], and a few others evaluated its psychometric properties in this population [10,11]. Similarly, the IES-2 has been applied during pregnancy [12,13,14] and translated into several languages [13,15,16,17,18,19,20,21,22,23,24]. However, to our knowledge, the IES-2 has not yet been translated and adapted into Spanish, and nor have its psychometric properties been evaluated in pregnant women.

In this study, our aim was to (1) translate and adapt the IES-2 to Mexican Spanish; (2) evaluate the psychometric properties of this adaptation with the hypothesis that it would be reliable for use with Mexican pregnant women; and (3) examine correlations among the four intuitive eating traits and maternal sociodemographic characteristics.

## 2. Materials and Methods

### 2.1. Scale Adaptation

The IES-2 original English version [6] underwent translation and cultural adaptation into Mexican Spanish by two nutrition specialists (GR and RVS). This translation can be found in Supplementary Table S1. A bilingual nutrition specialist, not part of the team, performed a back-translation of our adapted questionnaire to ensure that the items’ original meaning was unaltered. Approval for the study was granted by the Research and Ethics Committees of the National Institute of Perinatology (INPer) in Mexico City (Registration No. 2018-1-169).

### 2.2. Assessment of Psychometric Properties

#### 2.2.1. Participants and Procedures

Our adjusted IES-2 questionnaire was administered to 514 pregnant women receiving prenatal care at INPer who had consented to participate. To guarantee completion and secure storage of responses, we designed an online form using Google Forms. Participants received a unique link to access the form and complete the questionnaire electronically.

In addition to the IES-2 questionnaire, in the online form we asked participants about their age, number of pregnancies (including the current one), history of miscarriages or stillbirths, current gestational age, type of pregnancy (single or multiple), existing medical conditions (whether acute, like infections, or chronic, such as diabetes, autoimmune diseases, or thyroid conditions), highest education level, occupation (stay-at-home or work outside the home), cohabitation with the baby’s father, household socioeconomic status, height, and pregestational weight. The last two variables were used to calculate each woman’s pregestational body mass index (pg-BMI).

The “current illness” variable indicated the presence of acute or chronic medical conditions. Household welfare was evaluated with the AMAI 8x7 tool developed by the Mexican Association of Market Intelligence and Public Opinion Agencies (AMAI) [25], which categorizes households into seven socioeconomic levels (A/B to E). These categories were grouped into “Medium to High” (C+, C, C−, A/B) and “Low” (D+, D, E).

Upon collecting responses, a database was constructed for analyses, with participant anonymity maintained through unique ID numbers, accessible only to the principal investigator.

#### 2.2.2. Reliability and Factor Analyses

Descriptive measures, frequency distributions, and intra-class correlations were obtained for each IES-2 adaptation item to assess quality and viability, eliminating those with weak technical properties. To test whether items correctly discriminated between extreme answer groups, we compared those with the lowest scores on the total scale (below quartile 1) to those with the highest scores (above quartile 3) using Student *t*-tests for independent samples, considering *p* values ≤ 0.05 as significant.

To analyze construct validity, we first performed an exploratory principal component analysis with varimax rotation (Kaiser normalization) on the initial 23 items. We removed those items that loaded on two factors simultaneously (see Results section) and analyzed reliability in the remaining items with the Cronbach alpha test.

After these modifications, we conducted a confirmatory factor analysis aiming at a model fit with a Chi-square value (CMIN) between 1 and 3; Goodness of Fit Index (GFI) and baseline Comparative Fit Index (CFI) values approaching 1; and Root Mean Square Error of Approximation (RMSEA) values ranging from 0.05 to 0.08 (acceptable), ideally ≤ 0.05 [26].

Reliability and exploratory analyses were carried out in SPSS 20 (IBM) while the confirmatory factor analysis was conducted in AMOS version 23.

### 2.3. Intuitive Eating and Sociodemographic Variables

To explore the relationship between intuitive eating traits and maternal socio demographic and reproductive variables, and to evaluate how much the latter explained each trait’s variance, we performed (1) bivariate correlation (Spearman) tests and (2) multivariable linear regressions with backward variable selection (F significance: entry = 0.05/removal = 0.10), with each trait as the dependent variable. Collinearities between variables in the final models had a tolerance ≥0.589 and a variance inflation factor ≤1.62. We conducted these analyses in SPSS version 26, considering *p*-values ≤ 0.05 as significant.

## 3. Results

### 3.1. Psychometric Properties of the Adapted Scale

#### 3.1.1. Participants’ Characteristics

The sociodemographic characteristics of the 514 participating pregnant women are shown in Table 1, together with their median scores in the whole IES-2 and its sub-scales. Most participants (75.34%) were residents of Mexico City’s metropolitan area, multiparas, with single pregnancies (94.5%), high-school education or below (70.7%), stayed at home (68.9%), did not live with the baby’s father (64%), and lived in a low-welfare household (74.3%).

#### 3.1.2. Reliability Tests and Factor Analyses

From the responses provided by participants, we initially obtained descriptive statistics for each item’s responses to evaluate their quality and viability. We aimed to eliminate items exhibiting weak technical properties. The responses to the 23 items, adapted from the original scale, displayed comparable means closely aligned with the theoretical mean, and consistent and similar standard deviations (Supplementary Table S2). Participants’ responses spanned the entire Likert scale range (1 to 5), with normally distributed frequency distributions. No floor/ceiling effects were detected.

In an intra-class correlation analysis conducted to explore potential relationships between items, most correlation coefficients were below 0.20, indicating a lack of substantial correlation among them and suggesting they assess distinct constructs. Although some items exhibited correlations, none were eliminated at this stage (Supplementary Table S3).

Reliability analysis for the 23 items yielded a Cronbach value of α = 0.755. In light of this result, to enhance reliability, we removed items 1, 9, and 17; this led to an increased α value of 0.791.

Next, we evaluated the items’ discriminative capacity by comparing groups with extreme responses, those with the lowest scores on the total scale (<Q1, *n* = 129, 25.1%) versus the highest scores (>Q3, *n* = 124, 24.1%). The results indicated significant differences (*p* < 0.001) between these groups in every case, confirming their satisfactory discriminative capability.

We then assessed construct validity in the remaining 20 items through an exploratory factor analysis. Convergence after twenty iterations revealed five factors with eigenvalues greater than 1, collectively explaining 64.6% of the total variance (Supplementary Table S4). The analysis showed a Kaiser–Meyer–Olkin (KMO) value of 0.797, with a statistically significant Bartlett sphericity value (X^2^_(190)_ = 4261.444, *p* < 0.001).

**Figure 1:**
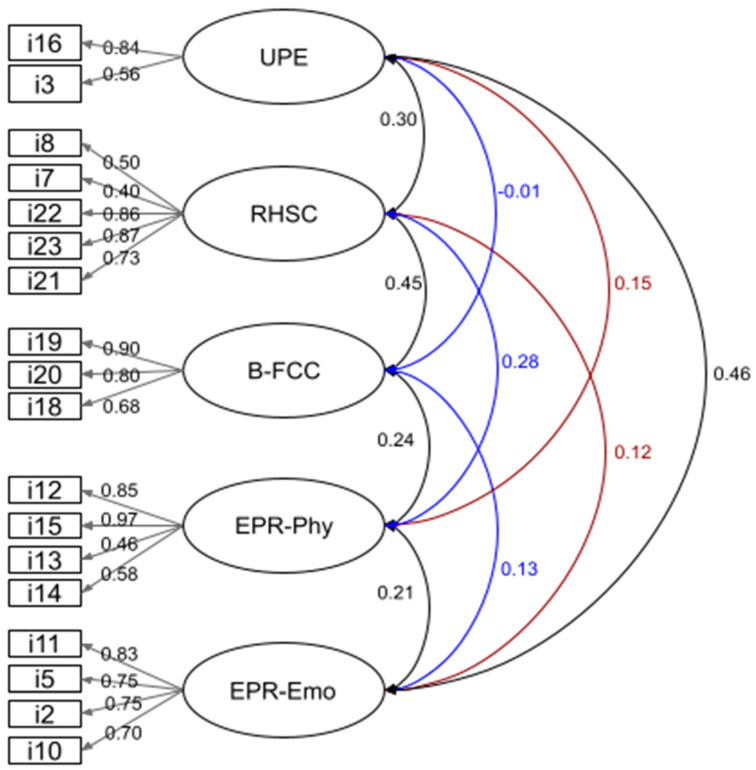
Confirmatory factor analysis. Numbers represent factor loads for the 18 items in the adapted version of the IES-2 (**left**) and inter-factor correlations (**right**). RHSC = Reliance on Hunger and Satiety Cues. EPR-Emo = Eating for Physical Rather Than Emotional Reasons (emotional component). B-FCC = Body–Food Choice Congruence. EPR-Phy = Eating for Physical Rather Than Emotional Reasons (physical component). UPE = Unconditional Permission to Eat.

Finally, we performed a confirmatory factor analysis. Following the proposed modifications to the model, we eliminated two additional items: item 6 which resulted in many correlated errors and item 4 which showed a very low factorial weight (0.15). Once these modifications were made, a good model fit was obtained as shown by the corresponding indices (CMIN = 1.99; GFI = 0.951; CFI = 0.968; RMSEA = 0.044, *p* = 0.883). The remaining 18 items loaded significantly on their respective factors with standardized parameters between 0.40 and 0.90 (Figure 1).

As observed in both exploratory and confirmatory factor analyses, an important difference from the original IES-2 is that our adapted version resulted in five factors, instead of the four from the original scale. The Unconditional Permission to Eat (UPE), Reliance on Hunger and Satiety Cues (RHSC), and Body–Food Choice Congruence (B-FCC) factors were conserved in our adaptation. However, the answers corresponding to items in the Eating for Physical rather than emotional Reasons (EPR) factor converged into two factors. 

The first factor contained items 2, 5, 10, and 11, which evaluate how much of the individual’s eating is related to emotional reasons within the EPR construct. The second factor contained items 12, 13, 14, and 15, which in contrast evaluate a more physical component in the EPR construct. This difference between our adaptation having five factors and the original four-factor scale is likely to be related to the fact that our adaptation was evaluated in pregnant women, in which the emotional component of experience would clearly be accentuated. We will delve into this topic in the Discussion section.

The final adapted scale, comprising 18 items grouped into five factors, is shown in Table 2.

### 3.2. Associations and Regression Analysis among Intuitive Eating Traits, and with Psychosocial and Reproductive Variables

Bivariate associations among intuitive eating traits are shown in Table 3. RHSC had a positive correlation with UPE, BFCC, and EPR-Ph. BFCC is also correlated with EPR-Ph and EPR-Emo. EPR-Emo correlated with EPR-Ph and negatively with UPE.

Our bivariate and multivariate analyses (Table 3 and Table 4, respectively) point out how sociodemographic and reproductive factors influence intuitive eating traits. In the bivariate correlations, maternal age and pg-BMI were associated with most traits and the overall IES-2 score, followed by schooling, having a twin pregnancy, and occupation. Notably, however, while every other factor showed different correlation patterns, pg-BMI was always negatively associated with intuitive eating traits. Similarly, in the multivariate model, having a twin pregnancy and higher household welfare were the most positively influencing factors on the overall IES-2 score, while pg-BMI had a negative influence on intuitive eating. Maternal age, gestational weeks, and having a current illness also showed statistical significance in the multivariate model; they may be considered as negligible due to their extremely low influence (β). It is important to underscore that, in the multivariate model, the aforementioned influences of sociodemographic and reproductive variables considered in our study explained only a very small proportion of the overall intuitive eating variance, as shown by the R^2^ and adjusted R^2^ scores.

## 4. Discussion

In this study, we describe the psychometric properties of a Mexican Spanish translation and adaptation of the IES-2. We also show how intuitive eating is influenced by maternal sociodemographic characteristics. This is the first time the scale has been translated and adapted into Spanish. Being culturally adapted to and with linguistic nuances of Latin American Spanish, it is appropriate for use with Latin American populations, particularly Mexican individuals. Such an adaptation, however, could represent a limitation when used with Spanish-speaking women from other regions; this would need to be tested in particular contexts. Furthermore, the psychometric structure and reliability of the adapted IES-2 were tested in pregnant women. These properties have already been tested in pregnant women but only for the first version of the scale [10], not for the IES-2, which we present here.

Our adaptation presents some differences from the original IES-2. First, it is composed of 18 items out of the 23 that comprise the original English version of the scale [6]. Five items (#1, 4, 6, 9, and 17) were eliminated in our adaptation; their elimination, however, did not compromise the instrument’s reliability as shown by psychometric analyses. The eliminated items were:

1. I try to avoid certain foods high in fat, carbohydrates, or calories.

4. I get mad at myself for eating something unhealthy.

6. I trust my body to tell me when to eat.

9. I have forbidden foods that I don’t allow myself to eat.

17. I do NOT follow eating rules or dieting plans that dictate what, when, and/or how much to eat.

Items 1, 9, and 17 were eliminated to increase the overall reliability value of the scale; they correspond to the Unconditional Permission to Eat (UPE) and Body–Food Choice Congruence (B-FCC) subscales. Items 4 and 6 were eliminated during the confirmatory factor analysis since they showed correlation errors or very low factorial weights; they correspond to the UPE and the Reliance on internal Hunger/Satiety Cues (RHSC) subscales. 

Removed items 1, 4, and 9, which correspond to the UPE subscale, are related to restrictive behaviors and prohibited foods. Previous research on different cultural backgrounds has documented that pregnant women spontaneously reduce restrictive eating practices and give themselves greater permission to eat [11,27]. Therefore, during pregnancy, these items may become neutral and thus not useful for recognizing UPE in this population. For example, English women have been shown to exhibit lower levels of restrained eating during the second trimester of pregnancy compared to a group of non-pregnant controls and to their own eating behavior three months before conception [27]. Nearly half of the pregnant participants in that study (42%) rated themselves as overeating, feeling they were allowed to eat more than they would usually eat, and indulging in what they usually thought of as “forbidden food”. Similarly, in a qualitative study in which twelve pregnant women in New Zealand were interviewed to test the content validity of the IES, some of the interviewees reported being less avoidant and feeling less guilty when eating foods with higher caloric, fat, or carbohydrate content compared to their pre-pregnant state [11]. Conversely, items 3 and 16, which remained in the UPE subscale, are related to permissive behaviors including the concept of “craving”, which is a common and accepted conduct among pregnant women [28,29].

In our version of the scale, it seems that the meaning of the UPE subscale has been subtly changed. The UPE scale was designed to assess the “individuals’ willingness to eat when hungry and the refusal to label certain foods as “forbidden”” [24]. However, in our adaptation, the items asking about prohibited foods have been eliminated and the remaining two items in the subscale do not distinguish whether the individual eats out of hunger, craving, or simply allows themself to eat when desired, possibly responding to social beliefs such as “eating for two during pregnancy”. A woman’s practice of restraining or eliminating certain foods during pregnancy may not be solely due to a restrictive behavior based on weight concern but may also be responding to other reasons. For example, some dietary guidelines recommend avoiding or reducing the intake of certain foods or beverages during pregnancy due to food safety concerns, such as alcohol and caffeine; raw or undercooked meat; poultry, seafood or eggs; unpasteurized milk; or soft cheeses [30]. There may also be cultural beliefs that discourage the intake of some foods [31,32]. Because of all these, when asking about forbidden foods or unconditional permission to eat, an IES aimed at pregnant women should consider these reasons separately. 

Paradoxically, removing item 17, originally in the B-FCC scale, suggests that at least some pregnant women may be willing to follow eating advice, rules, or dieting plans during this stage. This inference is supported by research reporting that some women during gestation make a conscious effort to modify what they eat [11,33]. These changes are motivated by (1) the baby’s health; (2) the mother’s health, especially if confronted with an increased risk of developing a nutrition-related disease such as gestational diabetes mellitus or high blood pressure; (3) weight gain management; and (4) food safety issues related to potential microbiological contamination [11,28,34]. Most often, women relate their motivation to the advice given by health professionals [33,34]. Although in our study we did not ask whether participants received nutrition information from INPer’s clinical personnel or other sources, it is likely that they did, since nutrition advice is a mandatory component of prenatal care in Mexico [35].

Items that remained in the B-FCC subscale were item 18 (“Most of the time, I desire to eat nutritious foods”), 19 (“I mostly eat foods that make my body perform efficiently (well)), and 20 (“I mostly eat foods that give my body energy and stamina”). These items denote “positive” characteristics of the diet according to what is usually recommended and expected from the diet by traditional health professionals at this stage. Nevertheless, being highly motivated to achieve a “healthy” diet does not always translate into adherence to recommendations, as shown by a study on pregnant Australian women [36].

The discussion about the modifications made to the UPE and B-FCC subscales may seem contradictory. On the one hand, some pregnant women relax their eating restrictions and stop limiting their food choices based on its energy, fat, or carbohydrate content. On the other hand, some women may be open to following rules and eating plans. This apparent discrepancy may be a sign of different attitudes among pregnant women towards what would be considered “healthy” eating from the traditional weight-centered approach to health. In a Norwegian study, some women experienced pregnancy as a “time-off” from eating “healthy” food, while others considered gestation a “turning point” towards a “healthier” diet [34]. However, we think these observations need a closer look, since, as mentioned earlier, motivations for restricting food and following eating rules may be different during pregnancy than for non-pregnant women. We suggest there is a need for further research, and possibly a need to incorporate new items in the UPE subscale that address different motivations for forbidding food. 

An important difference from the original scale is that, in our adaptation, the Eating for Physical rather than Emotional Reasons (EPR) subscale split into two factors (see Supplementary Table S4). The first factor comprises items 2 (“I find myself eating when I’m feeling emotional (e.g., anxious, depressed, sad), even when I’m not physically hungry”), 5 (“I find myself eating when I am lonely, even when I’m not physically hungry”), 10 (“I use food to help me soothe my negative emotions”), and 11 (“I find myself eating when I am stressed out, even when I’m not physically hungry”), which emphasize the emotional aspect of eating behavior, the very opposite of EPR. This is why the scores for these items should be reversed during the scale’s scoring procedure. We deemed this first factor as the “emotional component” of EPR (EPR-Emo). Conversely, the second factor into which the EPR subscale split was composed of items 12 (“I am able to cope with my negative emotions (e.g., anxiety, sadness) without turning to food for comfort”), 13 (“When I am bored, I do NOT eat just for something to do”), 14 (“When I am lonely, I do NOT turn to food for comfort”), and 15 (“I find other ways to cope with stress and anxiety than by eating”). These explore the EPR trait by emphasizing its non-emotional aspects. We deemed this factor to be the “physical component” of EPR (EPR-Phy).

To our knowledge, this division in the EPR subscale has not been reported for other IES-2 adaptations. We think this may be due to two reasons. First, the other studies in which the scale was adapted for pregnancy used its first version [10,11]. That version only included those items emphasizing the “emotional” component (i.e., those whose score should be reversed), but not the second set of items, the “non-emotional” ones; these were introduced in the second version of the scale (IES-2). This may be why the EPR subscale did not split into two factors when its psychometric properties were evaluated in pregnancy [10]. The second reason would be that studies applying the scale’s second version (IES-2) to pregnant women have not tested its psychometric properties in this population [12,13,14]; while those adaptations that did test psychometric properties did not include pregnant women [13,15,16,17,18,19,20,21,22,23,24]. Therefore, all existing adaptations so far have maintained the scale’s original structure.

An accentuation of the emotional component in spite of the physical component of the EPR subscale may not be entirely unexpected during pregnancy. This is a life period in which a wide range of emotional manifestations and potential stressors are present, nuanced by a woman’s personality and coping mechanisms. Emotions during pregnancy often manifest in ambivalent or mixed ways, from mild mood changes to emotional disturbances which may include traits associated with anxiety or depression [37]. Although some of the latter may require adequate treatment, in most cases such emotional manifestations are temporary and expected during pregnancy and are thus deemed to be psychological or emotional distress [38].

Therefore, given the prevalence of pregnancy-related emotional manifestations, it hardly comes as a surprise that women with more intuitive eating behaviors during their non-gravid state (i.e., with a stronger EPR trait) may be more inclined to base such behavior on their emotional component during pregnancy. Further studies evaluating the psychometric properties of the IES-2 during pregnancy are required to evaluate whether our finding regarding the split of the EPR subscale is replicated in other population settings.

Regarding the correlations between the subscales, our Spanish adaptation of the IES-2 has similar results to psychometric analyses of other versions. Moderate associations between the RHSC and the UPE and B-FCC subscales have been observed previously in Greek, Canadian, US, Brazilian, and German adults [6,15,17,18,21]. As in our study, others did not find an association between the UPE and the B-FCC subscales [17,21], while some observed a negative association between these two subscale scores [6,15,18]. This absence or negative correlation is logical since UPE measures the disposition to eat any food at any moment according to desire, while the items in the BFCC scale imply a degree of awareness of the needs of the body and making corresponding decisions regarding food choices and eating patterns. Concerning the association between EPR and the other scales, it is difficult to compare with other studies due to the division, in our study, of the subscale into emotional and physical subcomponents. However, some of the associations we observed seem logical; for example, the positive association between B-FCC and EPR-Ph seems logical, since both subscales suggest trust in internal and physical cues and food choices to enhance the body’s functions and well-being.

Concerning the influence of sociodemographic and reproductive factors on the total IES-2 score, twin pregnancy, pg-BMI, household welfare, and having a current illness were the most influencing factors. Regarding the type of pregnancy, we did not find studies to back up our observation; however, we propose a hypothesis to explain our finding: it has been reported that women with twin pregnancies experience vomiting, nausea, poor appetite, early satiety, and physical discomfort more often than women with singleton pregnancy, due to an enlarged uterus [39,40]. These physical sensations may trigger increased awareness of hunger and satiety cues and thus higher intuitive eating scores. 

With respect to pg-BMI, we observed a negative influence on the overall IES-2 scale score. Other studies have also documented a higher level of intuitive eating with lower BMI [6,15,16,17,20,22,41,42]. Among the postulated explanations for this inverse relationship is the hypothesis that the interoception and trust in hunger and satiety cues and less reliance on emotional or situational motivators to eat which are commonly observed with intuitive eating, positively influence food habits and the quantities of food consumed [43]. 

Household welfare had a positive influence on intuitive eating, as has been observed in previous studies [44,45]. In these, the authors discuss that intuitive eating might be difficult to practice within families who face structural inequities which prevent them from having continuous access to a variety of nutritious foods. For example, eating as much as possible and bypassing feelings of fullness might be an expected response in someone who is experiencing insecure access to food due to economic reasons. 

Lastly, having no baseline illness was associated with the overall IES-2 score. However, due to the nature of our variable, in which we grouped together all types of illness, it is difficult to discuss such an association. Some studies propose that BMI may be a mediator between intuitive eating and some physical health indicators such as blood pressure, cholesterol, and glucose levels [46,47], associated with some of the health issues observed in our study population. However, further research is needed to explain how other illnesses may influence intuitive eating behaviors.

Nevertheless, as mentioned earlier, it must be kept in mind that the influences of these sociodemographic and reproductive variables on intuitive eating were extremely low, explaining a very small proportion of the IES-2 score. This indicates that other variables, possibly of a psychological nature, that were not considered in this study play a more significant role in explaining the variation in the IES-2 and its subscales.

## 5. Conclusions

We present the first Spanish version of the IES-2 scale, which accounts for linguistic and cultural nuances used in Mexico. Its psychometric evaluation confirms the scale’s reliability and validity for its use with pregnant women. Our adaptation moves forward in the study of intuitive eating during pregnancy in two main ways. First, it underscores the importance of women’s emotional states on intuitive eating, particularly the EPR trait. Second, it highlights the need for further research into the concept and evaluation of the UPE trait during pregnancy, due to the removal and subtle changes in the meaning of some UPE items. A newer version of the scale should take into account pregnancy-related behavioral issues, such as the reasons why women consider some foods “forbidden” or the motivations to give themselves (or not) unconditional permission to eat.

## Figures and Tables

**Table 1:** Participants’ characteristics (*n* = 514).

	Median (Quartile 1–Quartile 3)
Maternal age (years)	29.7 (25.0–33.8)
Gestational age (weeks)	26.0 (21.0–31.0)
Pregestational BMI (kg/m^2^)	25.9 (23.1–30.1)
IES-2 total score	3.5 (3.2–3.8)
IES-2 Unconditional Permission to Eat	3.5 (3.0–4.0)
IES-2 Reliance on Hunger and Satiety Cues	3.6 (3.0–4.0)
IES-2 Body–Food Choice Congruence	3.7 (3.0–4.0)
IES-2 Eating for Physical Rather Than Emotional Reasons, physical component	3.5 (2.7–4.0)
IES-2 Eating for Physical Rather Than Emotional Reasons, emotional component	4.0 (3.2–4.5)
	*n* (%)
Number of pregnancies	
First pregnancy	199 (38.7)
2nd or 3rd	216 (42.0)
4th or more	99 (19.3)
Previous miscarriages/stillbirths	194 (37.7)
Singleton pregnancy	486 (94.5)
Living with a current illness	237 (46.1)
Schooling	
Elementary school	19 (3.7)
Secondary school	118 (23.0)
High school	226 (44.0)
Undergraduate school	124 (24.1)
Graduate school	27 (5.3)
Occupation	
Stays at home *	354 (68.9)
Works outside home	160 (31.1)
Lives with the baby’s father	
Yes	328 (64.0)
Household welfare	
Medium to high	132 (25.7)
Low	382 (74.3)

* Stays at home category includes students. Continuous variables’ groups compared with the Mann–Whitney test, categorical variables with the Fisher or Chi-square test.

**Table 2:** The 18-item Intuitive Eating Scale version 2, adapted to Mexican Spanish.

Factor	Item
Permiso incondicional para comer (Unconditional Permission to Eat)	3. Si tengo antojo de cierto alimento me doy permiso de comerlo. 16. Me doy permiso de comer cualquier comida que desee en el momento.
Confianza en las señales de hambre/saciedad (Reliance on Hunger and Satiety Cues)	7. Confío en que mi cuerpo me dice qué debo comer. 8. Confío en que mi cuerpo me dice cuánto debo comer. 21. Confío en mis señales de hambre para saber cuándo comer. 22. Confío en mis señales de llenado (saciedad) para saber cuándo dejar de comer. 23. Confío en mi cuerpo para saber cuándo dejar de comer.
Congruencia en la elección cuerpo-comida (Body–Food Choice Congruence)	18. La mayoría de las veces deseo comer alimentos nutritivos. 19. Principalmente como alimentos que hacen que mi cuerpo funcione eficientemente (bien). 20. Principalmente como alimentos que le dan a mi cuerpo energía y aguante.
Comer por razones físicas en vez de emocionales (componente físico). (Eating for Physical Rather Than Emotional Reasons, physical component)	12. Soy capaz de sobrellevar mis emociones negativas (ansiedad, tristeza) sin recurrir a la comida para sentirme mejor. 13. Cuando estoy aburrida NO como solamente para tener algo que hacer. 14. Cuando me siento sola NO recurro a la comida para sentirme mejor. 15. Encuentro otras formas de sobrellevar el estrés y la ansiedad que comiendo.
Comer por razones físicas en vez de emocionales (componente emocional). (Eating for Physical Rather Than Emotional Reasons, emotional component)	2. Me doy cuenta de que como cuando me siento emocional (ansiosa, deprimida, triste), aunque no tenga hambre. 5. Me doy cuenta de que como cuando me siento sola, aunque no tenga hambre. 10. Uso la comida para ayudarme con mis emociones negativas. 11. Me doy cuenta de que como cuando estoy estresada, aunque no tenga hambre.

**Table 3:** Correlations between intuitive eating traits and maternal sociodemographic factors.

	IES-2	UPE	RHSC	B-FCC	EPR-Ph	EPR-Emo	Maternal Age	pg-BMI	Number of Pregnancies	Previous Miscarriages	Type of Pregnancy	Gestational Age	Current Illness	Schooling	Occupation	Lives with Baby’s Father	Household Welfare
IES-2	_	_	_	_	_	_	0.042 (0.343)	**−0.182** **(<0.001)**	−0.038 (0.390)	0.003 (0.946)	**0.149** **(0.001)**	−0.063 (0.156)	−0.057 (0.196)	0.058 (0.190)	0.065 (0.138)	0.069 (0.121)	0.053 (0.234)
UPE		-	**0.248** **(<0.001)**	−0.047 (0.292)	0.078 (0.079)	**−0.148** **(0.001)**	**−0.090** **(0.043)**	**−0.134** **(0.002)**	−0.034 (0.442)	0.009 (0.833)	0.072 (0.105)	0.018 (0.678)	−0.078 (0.079)	0.031 (0.483)	0.021 (0.642)	0.003 (0.946)	−0.007 (0.872)
RHSC			-	**0.360** **(<0.001)**	**0.222** **(<0.001)**	0.064 (0.148)	0.017 (0.698)	**−0.221** **(<0.001)**	0.004 (0.927)	0.032 (0.466)	**0.124** **(0.005)**	−0.014 (0.758)	−0.050 (0.254)	0.023 (0.600)	0.025 (0.572)	0.056 (0.203)	0.043 (0.330)
B-FCC				-	**0.175** **(<0.001)**	**0.114** **(0.010)**	**0.155** **(<0.001)**	**−0.148** **(0.001)**	0.021 (0.631)	−0.026 (0.563)	**0.098** **(0.026)**	−0.031 (0.483)	0.077 (0.080)	0.056 (0.204)	−0.017 (0.696)	0.057 (0.193)	0.082 (0.062)
EPR-Ph					-	**0.210** **(<0.001)**	**0.121** **(0.006)**	−0.030 (0.501)	−0.039 (0.376)	0.023 (0.604)	0.077 (0.081)	−0.070 (0.111)	−0.038 (0.388)	**0.136** **(0.002)**	**0.134** **(0.002)**	0.058 (0.191)	0.073 (0.098)
EPR-Emo						-	**−0.090** **(0.042)**	−0.033 (0.457)	−0.010 (0.818)	−0.005 (0.901)	0.034 (0.445)	−0.062 (0.164)	−0.048 (0.275)	**−0.125** **(0.004)**	−0.007 (0.882)	0.020 (0.648)	−0.030 (0.500)

Spearman’s Rho correlations, *p* values in parentheses. Bold numbers indicate significant correlations. IES-2 = IES-2 total scale. UPE = Unconditional Permission to Eat. RHSC = Reliance on Hunger and Satiety Cues. B-FCC = Body–Food Choice Congruence. EPR-Ph = Eating for Physical Rather Than Emotional Reasons, Physical component. EPR-Emo = Eating for Physical Rather Than Emotional Reasons, Emotional component. pg-BMI = pregestational Body Mass Index. Number of pregnancies: first pregnancy = 1; second or third pregnancy = 2; fourth or more pregnancies = 3. Previous miscarriages/stillbirths: no = 0, yes = 1. Type of pregnancy: singleton = 1, multiple (two or more babies) = 2. Current illness: no = 0, yes = 1. Schooling: Elementary school = 1; Secondary school = 2; High school = 3; Undergraduate school = 4; Graduate school = 5. Occupation: stays at home = 1; works away from home = 2. Lives with the baby’s father: no = 0; yes = 1. Household welfare: low = 1; medium to high = 2.

**Table 4:** Linear regression model.

Total IES Score	
Predictors	β (95% CI) *p*-Value
Type of pregnancy	0.295 (0.121, 0.470) 0.001
Household welfare	0.123 (0.011, 0.236) 0.032
Maternal age	0.009 (0.002, 0.016) 0.013
Gestational weeks	−0.006 (−0.012, 0.000) 0.040
Current illness	−0.102 (−0.202, −0.003) 0.044
pg-BMI	−0.18 (−0.015, −0.011) <0.001
R^2^ (adjusted R^2^)	0.087 (0.076)

Type of pregnancy: singleton = 1, multiple = 2. Household welfare: low = 1, medium to high = 2. Current illness: no = 0; yes = 1. pg-BMI = pregestational Body Mass Index.

## Data Availability

The data presented in this study are available on request from the corresponding author.

## Supplementary Materials

The following supporting information can be downloaded at: https://www.mdpi.com/article/10.3390/nu15224837/s1, Table S1: Translation and adaptation of the Intuitive Eating Scale-2 into Mexican Spanish; Table S2: Descriptive statistics for responses to the 23 translated IES-2 items; Table S3: Item intra-class correlations; Table S4: Rotated component matrix.
