# Supplementary material for: The Intuitive Eating Scale-2 Adapted for Mexican Pregnant Women: Psychometric Properties and Influence of Sociodemographic Variables
Source: Nutrients. 2023 Nov 19;15(22):4837. doi: 10.3390/nu15224837 (PMC10675059; doi:10.3390/nu15224837)
Supplement: Supplementary file 1 [file nutrients-15-04837-s001.zip › nutrients-2693601-supplementary.pdf]

Supplementary Material

The Intuitive Eating Scale-2 adapted for Mexican pregnant women: psychometric properties and influence of sociodemographic variables.

Table S1. Translation and adaptation of the Intuitive Eating Scale-2 into Mexican Spanish.

**Original text *** (Tylka & Kroon Van Diest, J Couns Psych, 2013)	**Translated and adapted text ****
1. I try to avoid certain foods high in fat, carbohydrates, or calories.	1. Trato de evitar ciertos alimentos altos en grasa, carbohidratos o calorías.
2. I find myself eating when I'm feeling emotional (e.g., anxious, depressed, sad), even when I'm not physically hungry.	2. Me doy cuenta de que como cuando me siento emocional (ansiosa, deprimida, triste), aunque no tenga hambre.
3. If I am craving a certain food allow myself to have it.	3. Si tengo antojo de cierto alimento, me doy permiso de comerlo.
4. I get mad at myself for eating something unhealthy.	4. Me enojo conmigo misma cuando como algo no saludable.
5. I find myself eating when I am lonely, even when I'm not physically hungry.	5. Me doy cuenta de que como cuando me siento sola, aunque no tenga hambre.
6. I trust my body to tell me when to eat.	6. Confío en que mi cuerpo me dice cuándo debo comer.
7. I trust my body to tell me what to eat.	7. Confío en que mi cuerpo me dice qué debo comer.
8. I trust my body to tell me how much to eat.	8. Confío en que mi cuerpo me dice cuánto debo comer.
9. I have forbidden foods that I don't allow myself to eat.	9. Tengo alimentos prohibidos que no me permito comer.
10. I use food to help me soothe my negative emotions.	10. Uso la comida para ayudarme con mis emociones negativas.
11. I find myself eating when I am stressed out, even when I'm not physically hungry.	11. Me doy cuenta de que como cuando estoy estresada, aunque no tenga hambre.
12. I am able to cope with my negative emotions (e.g., anxiety, sadness) without turning to food for comfort.	12. Soy capaz de sobrellevar mis emociones negativas (como ansiedad, tristeza) sin recurrir a la comida para sentirme mejor.
13. When I am bored, I do NOT eat just for something to do.	13. Cuando estoy aburrida, NO como solamente para tener algo que hacer.
14. When I am lonely, I do NOT turn to food for comfort.	14. Cuando me siento sola, NO recurro a la comida para sentirme mejor.
15. I find other ways to cope with stress and anxiety than by eating.	15. Encuentro otras formas de sobrellevar el estrés y la ansiedad que comiendo.
16. I allow myself to eat what food I desire at the moment.	16. Me doy permiso de comer cualquier comida que desee en el momento.
17. I do NOT follow eating rules or dieting plans that dictate what, when, and/or how much to eat.	17. NO sigo reglas de alimentación o dietas que me dicen qué, cuándo y/o cuánto debo comer.

18. Most of the time, I desire to eat nutritious foods.	18. La mayoría de las veces deseo comer alimentos nutritivos.
19. I mostly eat foods that make my body perform efficiently (well).	19. Principalmente como alimentos que hacen que mi cuerpo funcione eficientemente (bien).
20. I mostly eat foods that give my body energy and stamina.	20. Principalmente como alimentos que le dan a mi cuerpo energía y aguante.
21. I rely on my hunger signals to tell me when to eat.	21. Confío en mis señales de hambre para saber cuándo comer.
22. I rely on my fullness (satiety) signals to tell me when to stop eating.	22. Confío en mis señales de llenado (saciedad) para saber cuándo dejar de comer.
23. I trust my body to tell me when to stop eating.	23. Confío en mi cuerpo para saber cuándo dejar de comer.

* Copyright © 2013 by American Psychological Association. Reproduced with permission. Tylka, T.L.; Kroon Van Diest, A.M. The Intuitive Eating Scale-2: Item Refinement and Psychometric Evaluation with College Women and Men. J. Couns. Psychol. 2013, 60, 137–153, doi:10.1037/a0030893.

** This material originally appeared in English as Tylka, T.L.; Kroon Van Diest, A.M. The Intuitive Eating Scale-2: Item Refinement and Psychometric Evaluation with College Women and Men. J. Couns. Psychol. 2013, 60, 137–153, doi:10.1037/a0030893. Copyright © 2013 by American Psychological Association. Translated and adapted with permission. American Psychological Association is not responsible for the quality or accuracy of this translation. This translation cannot be reproduced or distributed further without prior written permission.

Table S2. Descriptive statistics for responses to the 23 translated IES-2 items.

Translated item *	Subscale	Responses (n=514)			
		Mean	SD	Min.	Max.
1. Trato de evitar ciertos alimentos altos en grasa, carbohidratos o calorías.	UPE	2.77	0.93	1	5
2. Me doy cuenta de que como cuando me siento emocional (ansiosa, deprimida, triste), aunque no tenga hambre.	EPR	3.42	1.182	1	5
3. Si tengo antojo de cierto alimento, me doy permiso de comerlo.	UPE	3.71	0.856	1	5
4. Me enojo conmigo misma cuando como algo no saludable.	UPE	3.57	1.076	1	5
5. Me doy cuenta de que como cuando me siento sola, aunque no tenga hambre.	EPR	3.8	1.061	1	5
6. Confío en que mi cuerpo me dice cuándo debo comer.	RHSC	3.47	0.995	1	5
7. Confío en que mi cuerpo me dice qué debo comer.	RHSC	3.02	1.046	1	5
8. Confío en que mi cuerpo me dice cuánto debo comer.	RHSC	3.33	1.019	1	5
9. Tengo alimentos prohibidos que no me permito comer.	UPE	3.08	1.22	1	5
10. Uso la comida para ayudarme con mis emociones negativas.	EPR	4.1	0.875	1	5
11. Me doy cuenta de que como cuando estoy estresada, aunque no tenga hambre.	EPR	3.79	1.087	1	5
12. Soy capaz de sobrellevar mis emociones negativas (como ansiedad, tristeza) sin recurrir a la comida para sentirme mejor.	EPR	3.48	1.213	1	5
13. Cuando estoy aburrida, NO como solamente para tener algo que hacer.	EPR	3.06	1.232	1	5
14. Cuando me siento sola, NO recurro a la comida para sentirme mejor.	EPR	3.35	1.237	1	5
15. Encuentro otras formas de sobrellevar el estrés y la ansiedad que comiendo.	EPR	3.66	1.095	1	5
16. Me doy permiso de comer cualquier comida que desee en el momento.	UPE	3.21	1.016	1	5
17. NO sigo reglas de alimentación o dietas que me dicen qué, cuándo y/o cuánto debo comer.	UPE	2.82	1.105	1	5
18. La mayoría de las veces deseo comer alimentos nutritivos.	B-FCC	3.57	0.847	1	5
19. Principalmente como alimentos que hacen que mi cuerpo funcione eficientemente (bien).	B-FCC	3.62	0.798	1	5
20. Principalmente como alimentos que le dan a mi cuerpo energía y aguante.	B-FCC	3.61	0.783	1	5
21. Confío en mis señales de hambre para saber cuándo comer.	RHSC	3.68	0.851	1	5
22. Confío en mis señales de llenado (saciedad) para saber cuándo dejar de comer.	RHSC	3.72	0.884	1	5
23. Confío en mi cuerpo para saber cuándo dejar de comer.	RHSC	3.69	0.893	1	5

UPE=Unconditional Permission to Eat; EPR=Eating for Physical rather than emotional Reasons; RHSC=Reliance on internal Hunger/Satiety Cues; B-FCC=Body-Food Choice Congruence. * This material originally appeared in English as Tylka, T.L.; Kroon Van Diest, A.M. The Intuitive Eating Scale-2: Item Refinement and Psychometric Evaluation with College Women and Men. J. Couns. Psychol. 2013, 60, 137–153, doi:10.1037/a0030893. Copyright © 2013 by American Psychological Association. Translated and adapted with permission. American Psychological Association is not responsible for the quality or accuracy of this translation. This translation cannot be reproduced or distributed further without prior written permission.

Table S3. Item intra-class correlations.

Item number		1	2	3	4	5	6	7	8	9	10	11	12	13	14	15	16	17	18	19	20	21	22	23
**1**	r	1	0.060	0.201	0.191	-0.018	0.024	0.006	-0.030	0.312	-0.070	-0.064	-0.053	-0.184	-0.131	-0.150	0.318	0.331	-0.254	-0.261	-0.207	-0.032	-0.045	-0.060
	p		0.175	<0.001	<0.001	0.682	0.581	0.884	0.500	<0.001	0.112	0.148	0.227	<0.001	0.003	0.001	<0.001	<0.001	<0.001	<0.001	<0.001	0.467	0.314	0.173
**2**	r	0.060	1	-0.096	0.197	0.617	-0.026	-0.039	<0.001	0.028	0.480	0.625	0.214	-0.010	0.097	0.201	-0.071	-0.085	0.052	0.078	0.056	0.076	0.070	0.069
	p	0.175		0.029	<0.001	<0.001	0.551	0.383	0.994	0.524	<0.001	<0.001	<0.001	0.813	0.028	<0.001	0.109	0.054	0.239	0.077	0.205	0.087	0.110	0.119
**3**	r	0.201	-0.096	1	0.150	-0.081	0.195	0.103	0.175	0.165	-0.073	-0.147	0.052	0.034	0.031	0.029	0.478	0.180	-0.034	-0.026	-0.032	0.152	0.104	0.111
	p	<0.001	0.029		0.001	0.067	<0.001	0.019	<0.001	<0.001	0.100	0.001	0.236	0.444	0.488	0.509	<0.001	<0.001	0.437	0.553	0.473	0.001	0.018	0.012
**4**	r	0.191	0.197	0.150	1	0.270	0.033	0.014	0.050	0.306	0.246	0.231	0.050	-0.019	0.048	0.068	0.131	0.077	-0.097	-0.049	-0.031	0.037	0.019	0.049
	p	<0.001	<0.001	0.001		<0.001	0.449	0.745	0.259	<0.001	<0.001	<0.001	0.253	0.668	0.279	0.122	0.003	0.080	0.028	0.263	0.486	0.401	0.663	0.263
**5**	r	-0.018	0.617	-0.081	0.270	1	-0.021	-0.021	0.049	0.038	0.524	0.609	0.208	0.051	0.150	0.191	-0.104	-0.104	0.062	0.085	0.103	0.079	0.108	0.119
	p	0.682	<0.001	0.067	<0.001		0.629	0.637	0.264	0.393	<0.001	<0.001	<0.001	0.246	0.001	<0.001	0.018	0.019	0.158	0.053	0.019	0.073	0.014	0.007
**6**	r	0.024	-0.026	0.195	0.033	-0.021	1	0.532	0.578	-0.017	0.019	-0.076	0.182	0.080	0.157	0.217	0.253	0.131	0.155	0.173	0.158	0.473	0.365	0.430
	p	0.581	0.551	<0.001	0.449	0.629		<0.001	<0.001	0.703	0.674	0.085	<0.001	0.069	<0.001	<0.001	<0.001	0.003	<0.001	<0.001	<0.001	<0.001	<0.001	<0.001
**7**	r	0.006	-0.039	0.103	0.014	-0.021	0.532	1	0.652	-0.017	-0.056	-0.067	0.067	0.094	0.129	0.097	0.230	0.159	0.124	0.151	0.123	0.361	0.290	0.302
	p	0.884	0.383	0.019	0.745	0.637	<0.001		<0.001	0.705	0.204	0.130	0.130	0.033	0.003	0.029	<0.001	<0.001	0.005	0.001	0.005	<0.001	<0.001	<0.001
**8**	r	-0.030	<0.001	0.175	0.050	0.049	0.578	0.652	1	-0.082	0.032	-0.001	0.180	0.123	0.161	0.217	0.173	0.086	0.155	0.175	0.162	0.398	0.386	0.442
	p	0.500	0.994	<0.001	0.259	0.264	<0.001	<0.001		0.065	0.475	0.981	<0.001	0.005	<0.001	<0.001	<0.001	0.052	<0.001	<0.001	<0.001	<0.001	<0.001	<0.001
**9**	r	0.312	0.028	0.165	0.306	0.038	-0.017	-0.017	-0.082	1	0.075	0.019	-0.128	-0.158	-0.125	-0.162	0.243	0.191	-0.179	-0.190	-0.139	-0.045	-0.161	-0.083
	p	<0.001	0.524	<0.001	<0.001	0.393	0.703	0.705	0.065		0.089	0.665	0.004	<0.001	0.005	<0.001	<0.001	<0.001	<0.001	<0.001	0.002	0.306	<0.001	0.060
**10**	r	-0.070	0.480	-0.073	0.246	0.524	0.019	-0.056	0.032	0.075	1	0.659	0.259	0.073	0.151	0.281	-0.096	-0.166	0.083	0.141	0.175	0.182	0.200	0.202
	p	0.112	<0.001	0.100	<0.001	<0.001	0.674	0.204	0.475	0.089		<0.001	<0.001	0.097	0.001	<0.001	0.029	<0.001	0.059	0.001	<0.001	<0.001	<0.001	<0.001
**11**	r	-0.064	0.625	-0.147	0.231	0.609	-0.076	-0.067	-0.001	0.019	0.659	1	0.159	0.020	0.122	0.211	-0.180	-0.163	0.099	0.112	0.084	0.055	0.087	0.120
	p	0.148	<0.001	0.001	<0.001	<0.001	0.085	0.130	0.981	0.665	<0.001		<0.001	0.656	0.006	<0.001	<0.001	<0.001	0.024	0.011	0.056	0.211	0.049	0.007
**12**	r	-0.053	0.214	0.052	0.050	0.208	0.182	0.067	0.180	-0.128	0.259	0.159	1	0.363	0.522	0.544	0.066	-0.037	0.139	0.170	0.137	0.205	0.203	0.169
	p	0.227	<0.001	0.236	0.253	<0.001	<0.001	0.130	<0.001	0.004	<0.001	<0.001		<0.001	<0.001	<0.001	0.135	0.405	0.002	<0.001	0.002	<0.001	<0.001	<0.001
**13**	r	-0.184	-0.010	0.034	-0.019	0.051	0.080	0.094	0.123	-0.158	0.073	0.020	0.363	1	0.641	0.417	0.029	0.010	0.024	0.102	0.014	0.049	0.094	0.104
	p	<0.001	0.813	0.444	0.668	0.246	0.069	0.033	0.005	<0.001	0.097	0.656	<0.001		<0.001	<0.001	0.515	0.819	0.592	0.021	0.749	0.265	0.033	0.018
**14**	r	-0.131	0.097	0.031	0.048	0.150	0.157	0.129	0.161	-0.125	0.151	0.122	0.522	0.641	1	0.566	0.063	-0.043	0.065	0.147	0.105	0.108	0.162	0.141
	p	0.003	0.028	0.488	0.279	0.001	<0.001	0.003	<0.001	0.005	0.001	0.006	<0.001	<0.001		<0.001	0.157	0.328	0.143	0.001	0.018	0.014	<0.001	0.001
**15**	r	-0.150	0.201	0.029	0.068	0.191	0.217	0.097	0.217	-0.162	0.281	0.211	0.544	0.417	0.566	1	0.085	-0.029	0.168	0.213	0.220	0.248	0.279	0.257
	p	0.001	<0.001	0.509	0.122	<0.001	<0.001	0.029	<0.001	<0.001	<0.001	<0.001	<0.001	<0.001	<0.001		0.053	0.513	<0.001	<0.001	<0.001	<0.001	<0.001	<0.001
**16**	r	0.318	-0.071	0.478	0.131	-0.104	0.253	0.230	0.173	0.243	-0.096	-0.180	0.066	0.029	0.063	0.085	1	0.380	-0.069	-0.024	-0.028	0.227	0.177	0.191
	p	<0.001	0.109	<0.001	0.003	0.018	<0.001	<0.001	<0.001	<0.001	0.029	<0.001	0.135	0.515	0.157	0.053		<0.001	0.117	0.588	0.534	<0.001	<0.001	<0.001
**17**	r	0.331	-0.085	0.180	0.077	-0.104	0.131	0.159	0.086	0.191	-0.166	-0.163	-0.037	0.010	-0.043	-0.029	0.380	1	-0.075	-0.116	-0.092	0.106	0.052	0.085
	p	<0.001	0.054	<0.001	0.080	0.019	0.003	<0.001	0.052	<0.001	<0.001	<0.001	0.405	0.819	0.328	0.513	<0.001		0.089	0.009	0.038	0.016	0.244	0.053
**18**	r	-0.254	0.052	-0.034	-0.097	0.062	0.155	0.124	0.155	-0.179	0.083	0.099	0.139	0.024	0.065	0.168	-0.069	-0.075	1	0.618	0.490	0.295	0.306	0.261
	p	<0.001	0.239	0.437	0.028	0.158	<0.001	0.005	<0.001	<0.001	0.059	0.024	0.002	0.592	0.143	<0.001	0.117	0.089		<0.001	<0.001	<0.001	<0.001	<0.001
**19**	r	-0.261	0.078	-0.026	-0.049	0.085	0.173	0.151	0.175	-0.190	0.141	0.112	0.170	0.102	0.147	0.213	-0.024	-0.116	0.618	1	0.710	0.313	0.304	0.279
	p	<0.001	0.077	0.553	0.263	0.053	<0.001	0.001	<0.001	<0.001	0.001	0.011	<0.001	0.021	0.001	<0.001	0.588	0.009	<0.001		<0.001	<0.001	<0.001	<0.001

**20**	r	-0.207	0.056	-0.032	-0.031	0.103	0.158	0.123	0.162	-0.139	0.175	0.084	0.137	0.014	0.105	0.220	-0.028	-0.092	0.490	0.710	1	0.383	0.365	0.343
	p	<0.001	0.205	0.473	0.486	0.019	<0.001	0.005	<0.001	0.002	<0.001	0.056	0.002	0.749	0.018	<0.001	0.534	0.038	<0.001	<0.001		<0.001	<0.001	<0.001
**21**	r	-0.032	0.076	0.152	0.037	0.079	0.473	0.361	0.398	-0.045	0.182	0.055	0.205	0.049	0.108	0.248	0.227	0.106	0.295	0.313	0.383	1	0.620	0.623
	p	0.467	0.087	0.001	0.401	0.073	<0.001	<0.001	<0.001	0.306	<0.001	0.211	<0.001	0.265	0.014	<0.001	<0.001	0.016	<0.001	<0.001	<0.001		<0.001	<0.001
**22**	r	-0.045	0.070	0.104	0.019	0.108	0.365	0.290	0.386	-0.161	0.200	0.087	0.203	0.094	0.162	0.279	0.177	0.052	0.306	0.304	0.365	0.620	1	0.773
	p	0.314	0.110	0.018	0.663	0.014	<0.001	<0.001	<0.001	<0.001	<0.001	0.049	<0.001	0.033	<0.001	<0.001	<0.001	0.244	<0.001	<0.001	<0.001	<0.001		<0.001
**23**	r	-0.060	0.069	0.111	0.049	0.119	0.430	0.302	0.442	-0.083	0.202	0.120	0.169	0.104	0.141	0.257	0.191	0.085	0.261	0.279	0.343	0.623	0.773	1
	p	0.173	0.119	0.012	0.263	0.007	<0.001	<0.001	<0.001	0.060	<0.001	0.007	<0.001	0.018	0.001	<0.001	<0.001	0.053	<0.001	<0.001	<0.001	<0.001	<0.001	

r = Spearman correlation value; p = significance value.

Table S4. Rotated component matrix.

Item *		Factor			
	**1** **UPE**	**2** **RHSC**	**3** **B-FCC**	**4** **EPR-Phy**	**5** **EPR-Emo**
3. Si tengo antojo de cierto alimento, me doy permiso de comerlo.	0.777				
16. Me doy permiso de comer cualquier comida que desee en el momento.	0.758				
4. Me enojo conmigo misma cuando como algo no saludable.	0.490				0.425
8. Confío en que mi cuerpo me dice cuánto debo comer.		0.842			
7. Confío en que mi cuerpo me dice qué debo comer.		0.807			
6. Confío en que mi cuerpo me dice cuándo debo comer.		0.771			
23. Confío en mi cuerpo para saber cuándo dejar de comer.		0.636	0.410		
22. Confío en mis señales de llenado (saciedad) para saber cuando dejar de comer.		0.573	0.473		
21. Confío en mis señales de hambre para saber cuándo comer.		0.569	0.464		
19. Principalmente como alimentos que hacen que mi cuerpo funcione eficientemente (bien).			0.839		
20. Principalmente como alimentos que le dan a mi cuerpo energía y aguante.			0.837		
18. La mayoría de las veces deso comer alimentos nutritivos.			0.770		
14. Cuando me siento sola NO recurro a la comida para sentirme mejor.				0.867	
13. Cuando estoy aburrida NO como solamente para tener algo que hacer.				0.810	
15. Encuentro otras formas de sobrellevar el estrés y la ansiedad que comiendo.				0.736	
12. Soy capaz de sobrellevar mis emociones negativas (como ansiedad, tristeza) sin recurrir a la comida para sentirme mejor.				0.706	
11. Me doy cuenta de que como cuando estoy estresada aunque no tenga hambre.					0.842
5. Me doy cuenta de que como cuando me siento sola, aunque no tenga hambre.					0.811
2. Me doy cuenta de que como, cuando me siento emocional (ansiosa, deprimida, triste), aunque no tenga hambre.					0.802
10. Uso la comida para ayudarme con mis emociones negativas.					0.764

Rotation converged in 20 iterations. * This material originally appeared in English as Tylka, T.L.; Kroon Van Diest, A.M. The Intuitive Eating Scale-2: Item Refinement and Psychometric Evaluation with College Women and Men. J. Couns. Psychol. 2013, 60, 137–153, doi:10.1037/a0030893. Copyright © 2013 by American Psychological Association. Translated and adapted with permission. American Psychological Association is not responsible for the quality or accuracy of this translation. This translation cannot be reproduced or distributed further without prior written permission.
